# Identification of a novel human papillomavirus by metagenomic analysis of vaginal swab samples from pregnant women

**DOI:** 10.1186/s12985-016-0583-6

**Published:** 2016-07-04

**Authors:** Zhijian Liu, Shixing Yang, Yan Wang, Quan Shen, Yan Yang, Xutao Deng, Wen Zhang, Eric Delwart

**Affiliations:** School of Medicine, Jiangsu University, 301 Xuefu Road, Zhenjiang, Jiangsu 212023 People’s Republic of China; The Fourth Affiliated Hospital of Jiangsu University, 20 Zhengdong Road, Zhenjiang, Jiangsu 212001 China; Blood Systems Research Institute, Department of Laboratory Medicine, University of California San Francisco, San Francisco, CA 94118 USA; Department of Microbiology, School of Medicine, Jiangsu University, Zhenjiang, Jiangsu 212013 China

**Keywords:** Human papillomavirus, Virus metagenomics, Complete genome, Phylogenetic analysis

## Abstract

**Background:**

The number of members in the genus Gammapapillomavirus of Family Papillomaviridae has recently been expanding most rapidly. The aim of this study was to characterize a novel human gammapapillomavirus type identified in a vaginal swab from a 25-year-old pregnant woman suffering from vaginitis.

**Methods:**

Viral metagenomics method was used to detect the viral sequences in 88 vaginal swab samples collected from 88 pregnant women with vaginitis. A novel papillomavirus, named HPV-ZJ01 (GenBank no. KX082661), was detected in one sample and its complete genome sequence was amplified by PCR and sequenced by Sanger walking. Phylogenetic analyses based on the complete genome and the L1 protein of HPV-ZJ01 and other representative human papillomaviruses were done, respectively. Further PCR screening was performed in vaginal swabs (n = 135), cervical smears (n = 40) and cervical cancer tissues (n = 40) using nested-PCR primers designed based on HPV-ZJ01 sequence to investigate the prevalence of HPV-ZJ01.

**Results:**

The genome of HPV-ZJ01 is 7,358 bp in length with a GC content of 37.8 %. HPV-ZJ01 was predicted to contain six open reading frames (E6, E7, E1, E2, L2, and L1) and a non-coding long control region (LCR). The genome shared the highest overall similarity to HPV-166, with 70.6 % nucleotide sequence identity while its L1 gene shared the highest nucleotide similarity to HPV-162, with 71.1 % sequence identity. Phylogenetic analysis suggested that HPV-ZJ01 belongs to a novel HPV type in the Gamma-PV genus, species Gamma-19, already containing HPV161, HPV162 and HPV166. PCR screening results indicated that none of the other samples were positive for HPV-ZJ01 except the original HPV-ZJ01 positive vaginal swab specimen.

**Conclusion:**

The genome sequence of a novel type of species Gamma-19 HPV was characterized. The screening PCR results suggested that HPV-ZJ01 is not associated with any of the cervical cancer samples tested. In order to confirm the prevalence and disease association, if any, for HPV-ZJ01, a further study with different sample types and a larger sample size is needed.

## Background

Human papillomaviruses (HPVs) belong to family Papillomaviridae, with small, circular, double-stranded DNA genomes [1]. Papillomaviruses are highly diverse and infect human epithelial cells, where some species are implicated in the development of several benign and malignant neoplasms [1, 2]. Based on the nucleotide similarity in the L1 open reading frame (ORF), HPVs are classified into genera, species and types [1, 3]. HPV types that display less than 60 % nucleotide sequence similarity in the L1 open reading frame (ORF) are classified into different genera, while viral species share 60–70 % nucleotide sequence similarity, and types show at least 90 % [1]. The taxonomy of papillomaviruses at the species level and above is overseen by the International Committee for the Taxonomy of Viruses; while, HPV type designation and classification below the species level is maintained by the International HPV Reference Center at the Karolinska Institute, Stockholm, Sweden [4]. As of May 6, 2016, two hundred and one different HPV types, ranging from HPV-1 to HPV-205, were officially recognized, including 65 Alphapapillomaviruses (Alpha-PVs), 51 Betapapillomaviruses (Beta-PVs), 81 Gammapapillomaviruses (Gamma-PVs), 3 Mupapillomaviruses (Mu-PVs), and a single Nupapillomavirus (Nu-PV) [5]. Four previously recognized HPV types (HPV-46, HPV-55, HPV-64 and HPV-79) were recently re-classified as subtypes (HPV-79 was replaced by HPV91) [6–8].

As more HPV types are discovered, it is necessary to confirm the genomic characteristics and the clinical relevance of these new HPV types. Although a variety of laboratory techniques for detecting known HPVs are available, an efficient laboratory method for the identification of divergent HPVs from different biological samples is required.

Viral metagenomics is a useful method that can non-specifically detect both already known and highly divergent viruses. Non capsid protected host-derived background DNA and RNA in biological samples can be digested by nuclease treatment and nucleotide sequence information from viral particles associated nucleic acids determined by sequence-independent amplification and deep sequencing to allows the identification of viruses recognizable through their nucleotide or translated protein sequence homologies to all known viruses. With the advent of viral metagenomics [9–12] and the use of rolling circle amplification (RCA) which preferentially amplifies DNA circles [13, 14], it becomes possible to obtain the complete genome of new HPVs at relatively high speed.

In the present study, a viral metagenomic method was applied to vaginal swab samples collected from pregnant women, and identified a novel gamma papillomavirus in a sample from a 25-year-old pregnant woman suffering from vaginitis. We describe here the genome and phylogenetic analysis of this novel Gamma-PV type. Type-specific PCR primers were also developed to test a representative collection of various clinical specimens.

## Methods

### Sample collection and metagenomic analysis

From Jan. 2014 to Jan. 2015, 88 vaginal swab specimens were collected from 88 pregnant women with vaginitis in the affiliated Maternity and Infant Hospital of Jiangsu University, China. Swab specimens were re-suspended in 500ul of phosphate-buffered saline (PBS) and vigorously vortexed for five min. The suspension of each sample was subjected to centrifugation (10 min, 15,000 × *g*). The supernatant was filtered through a 0.45-μm filter (Millipore) to remove eukaryotic and bacterial cell-sized particles. The filtrates enriched in viral particles were treated with DNase and RNase to digest unprotected nucleic acid at 37 °C for 60 min [15]. Remaining total nucleic acid was then isolated using QiaAmp Mini Viral RNA kit (Qiagen) according to manufacturer’s protocol. Six separate pools were randomly generated, 5 of which contained nucleic acid from 15 specimens while one included 13 specimens. Six libraries were then constructed using Nextera XT DNA Sample Preparation Kit (Illumina) and sequenced using the MiSeq Illumina platform with 250 bases paired ends with dual barcoding for each pool. Bioinformatics analysis was performed according to a previous study [16].

### Finding and genome sequencing of the novel HPV

Viral metagenomics analysis indicated that one pool contained 30 sequence reads generating 11 different contigs ranging from 152 bp to 612 bp in length with amino acid homology to but significantly divergent from known HPV genomes in GenBank. PCR screening of the 15 individual samples in this pool with a set of nested primers designed on the 612 bp contig indicated that a single sample was positive. PCR to bridge sequence gaps were then used to acquire the whole genome of the novel HPV. PCR fragments were cloned in T-vectors and sequenced by Sanger walking. Sequences and characteristics of the primers used in PCR screening and genome determination are shown in Table 1.

**Table 1:** nested primers used to detect the positive sample of HPV-ZJ01 and amplify the gaps between contigs

Primers’ name	Sequence (5′to 3′)	Primers′ name	Sequence (5′to 3′)
Detection FO	AAATGAAACGCGGCAAGATCC	Primer4 FI	TGGCAAAAAGTGCAAGGAACTG
Detection RO	TCGGTTGCTCTTGGACTACG	Primer4 RI	AAGGCATACAGCATTCTACTCC
Detection FI	CGCGGCAAGATCCTGCAAATCA	Primer5 FO	AAGGGGACACATGGGCAGAT
Detection RI	GCTGTGTGCCTTCGTACCGGG	Primer5 RO	GCGGGAAACTGAGCCTAAGAA
Primer1 FO	GCTTGATGACTTTTGTTCTCACT	Primer5 FI	AGGTACTGGTAGGGGGTCTG
Primer1 RO	TAAGTGGACCTCCCATGCTG	Primer5 RI	CTTGGACAGGCGTGCTAGAT
Primer1 FI	AGGATCTTGCCGCGTTTCATT	Primer6 FO	TCTTAGGCTCAGTTTCCCGC
Primer1 RI	TAAGTGGACCTCCCATGCTG	Primer6 RO	AGTCTGTGGGCTTAATAGGCG
Primer2 FO	GTCAGCATGGGAGGTCCACT	Primer6 FI	CCGCCTTTGAACCAGATGTC
Primer2 RO	TGCTGCATTAGTATCAGTTTCTGC	Primer6 RI	CCAACCTGCAAGCCACTTCT
Primer2 FI	ATGAGAATGTGGATGCTGAGGA	Primer7 FO	TCTGCAATGACCACCAGAAGT
Primer2 RI	AGCCCACTGTATCATTCGAGAC	Primer7 RO	AGAGAACCACTAGGTGAAGCAA
Primer3 FO	GTGACAGATGTAAAACCGAAGG	Primer7 FI	GGCCTGTCCATTTTTATCAGGA
Primer3 RO	TGCCCATTTGAATAGCTTGCTT	Primer7 RI	CACTGGAAGGTGTTGAATTTCC
Primer3 FI	GTGACAGATGTAAAACCGAAGG	Primer8 FO	GAGAGCTCAAGGACCAAACAA
Primer3 RI	AGCTTGCTTAGCATTATTTTCAGAT	Primer8 RO	AAGGATGTCCAGATGTGACTG
Primer4 FO	GCCTTGCCTTATCCTTGTTGG	Primer8 FI	GAGAGCTCAAGGACCAAACAA
Prime4 RO	AGCACGTTTATTTCTACCAGCTC	Primer8 RI	TAACAAGCACCAAACAACTCCC

### PCR screening

In order to assess the prevalence of HPV-ZJ01 and whether this novel HPV strain was associated with cervical carcinoma, PCR was performed to screen the original 88 vaginal swab specimens, plus 128 samples from 128 patients suffering from cervical carcinoma, including 40 cancer tissues, 40 cervical smears, and 48 vaginal swab specimens, collected in 2014–2016 from the affiliated Maternity and Infant Hospital of Jiangsu University, China. All samples were frozen immediately at −80 °C until further processing. Before nucleic acid extraction, tissue samples were homogenized, frozen and thawed on dry ice three times. Nucleic acids were extracted by QiaAmp Mini Viral RNA kit (Qiagen). Two sets of PCR screening primers were designed based on the genome sequence of HPV-ZJ01 (Table 1). Standard precautions were used for all procedures to reduce the possibility of sample contamination by amplified DNA molecules. Negative and positive controls were included from nucleic acid extraction to PCR screening. The original HPV positive swab sample identified in this study was used as positive control. PBS was used as negative control instead of samples during nucleic acid extraction, while deionized water was used as negative control instead of nucleic acid template in PCR screening.

### Phylogenetic analysis

Phylogenetic analyses were performed including the closest viral relatives based on best BLASTx hits and representative members of related viral species and genera. Sequence alignment was performed using CLUSTALW with the default settings. A phylogenetic tree with 1000 bootstrap resamples of the alignment data sets was generated using the maximum likelihood method based on Jones-Taylor-Thornton (JTT) model in MEGA6.0. Bootstrap values (based on 1000 replicates) for each node are given. Putative ORFs in the genome were predicted by NCBI ORF finder. Informed consents were provided by all participants included in the present study. Ethical Approval was given by Ethics Committee of Jiangsu University and the reference number is No. JSU2015045.

### Nucleotide sequence accession numbers

The genome sequence of HPV-ZJ01 was deposited in GenBank under the accession number: KX082661.

## Results

### Genomic organization

**Fig. 1:**
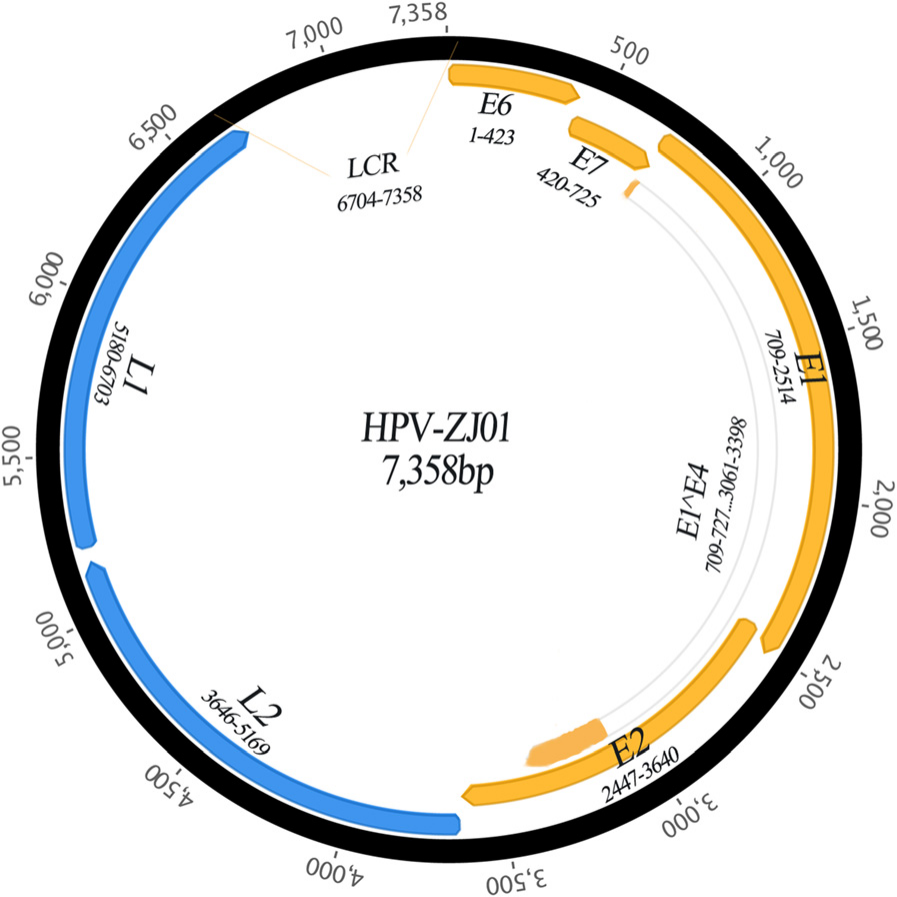
Genomic organization of HPV-ZJ01. Genomic positions of viral genes (E6, E7, E1, E2, E1^E4, L1 and L2) and the non-coding region (LCR) located between L1 and E6 are indicated

The complete circular genome of HPV-ZJ01 is 7,358 bp in length with a GC content of 37.8 %. The HPV-ZJ01 genome was predicted to contain six ORFs coding for four early proteins (E6, E7, E1, and E2) and two late proteins (L1 and L2) (Fig. 1). In addition, there was a 655 bp non-coding long control region (LCR), at position 6,704 through 7,358 of the genome, also known as an upstream regulatory region (URR) located between the L1 and E6 genes. The ‘A’ in the first ATG codon of the ORF E6 was assigned position 1 in the sequence. This LCR contains a putative TATA box (TATAAA, nucleotide positions 7,317-7,322), a putative polyadenylated site (AATAAA, nucleotide positions 6,929-6,934), three putative E1 binding sites (consensus sequence: AACAAT, nucleotide positions 6,799-6,804; 6,834-6,839 and 7,256-7,261), probably representing the origin of replication [13, 17], two palindrome sites (ACCG-N4-CGGT; nucleotide positions 7,080–7,091; 7,122–7,133) and three degenerate palindromes (ACC-N6/7-GGT, positions 6,795-6,807; 7,232-7,243 and 7,302-7,313). The palindrome motif ACCG-N4-CGGT is known to play a crucial role in binding of the E2 protein [18, 19], which mediates viral transcription in trans [20]. The putative E6 protein contains two conserved zinc-finger domains (CxxC(x)29CxxC) at positions 79–189, 298–408 and are separated by 36 amino acids. The putative E7 protein, located downstream of E6 ORF, contains only one zinc-finger domain at position 579–689 as well as the tumor suppressor (pRB) binding domain (LXCXE) at position 489–503 [21, 22]. One conserved ATP-binding site of the ATP-dependent DNA helicase (GPPDSGKS [G-X4-GKT/S]) was identified at position 1,993-2,016 in the putative E1 protein, encoded by the largest ORF (603 amino acid residues) [23]. No conserved leucine-zipper domain (L-X6-L-X6-L-X6-L), necessary for E2 dimerization, was observed in the carboxyterminal part of the putative E2 protein of HPV-ZJ01.

We identified the characteristic donor (AAG/GUASNR) and acceptor (GUYACYAG/YU) RNA splicing sites [13, 24–26] and it is likely that the E4 protein is translated from a spliced mRNA, containing the first few codons of the E1 ORF joined to the E4 sequence (Fig. 1, E1^E4, coding sequence consisting of genomic positions nt 709–727 and 3,061-3,398), as described previously for other HPV types [13, 24–26]. The resulting putative E1^E4 fusion protein contains a typically high content of proline, with 13 proline residues out of 118 amino acids (11 %).

### Sequence and phylogenetic analysis

Sequence analysis indicated that the L1 gene of the HPV-ZJ01 shares the highest nucleotide (nt) sequence identity (71.1 %) with HPV162, which was isolated from human healthy skin in China belonging to a member of the genus Gammapapillomavirus [27]. According to the International Committee on Taxonomy of Viruses (ICTV), the members of a papillomavirus species should share at least 70 % nt sequence identity over the L1 gene [3], it is therefore inferred that HPV-ZJ01 belongs into the Gamma-PV genus, species Gamma-19, already containing HPV161, HPV162 and HPV166. The nt sequence identities of L1 gene between HPV-ZJ01 and HPV161 and HPV166 were 69.1 % and 70.3 %, respectively. At the complete genome level, HPV-ZJ01 shares 69.5 %, 69.6 % and 70.6 % nt sequence identities with HPV161, HPV162 and HPV166, respectively. Table 2 shows the identities of the putative proteins of HPV-ZJ01 to the analogous proteins of HPV161, HPV162 and HPV166. Interestingly, the E6 and L2 proteins of HPV-ZJ01 are most closely related to those of HPV166, whereas the E1 and E2 proteins are closest to HPV161. E7 shares the same 64 % identity with HPV162 and HPV166 while L1 is closest to that of type 162, sharing 68.7 % amino acid sequence identity.

**Table 2:** Identity of HPV-ZJ01 Amino Acid Sequences with Related HPVs

HPV-ZJ01	E6	E7	E1	E2	L2	L1
HPV161	56.8 %	60.0	70.8	63.7	62.3	67.4
HPV162	58.3	64.0	69.8	62.6	62.6	68.7
HPV166	59.7	64.0	70.7	61.1	62.9	68.3

**Fig. 2:**
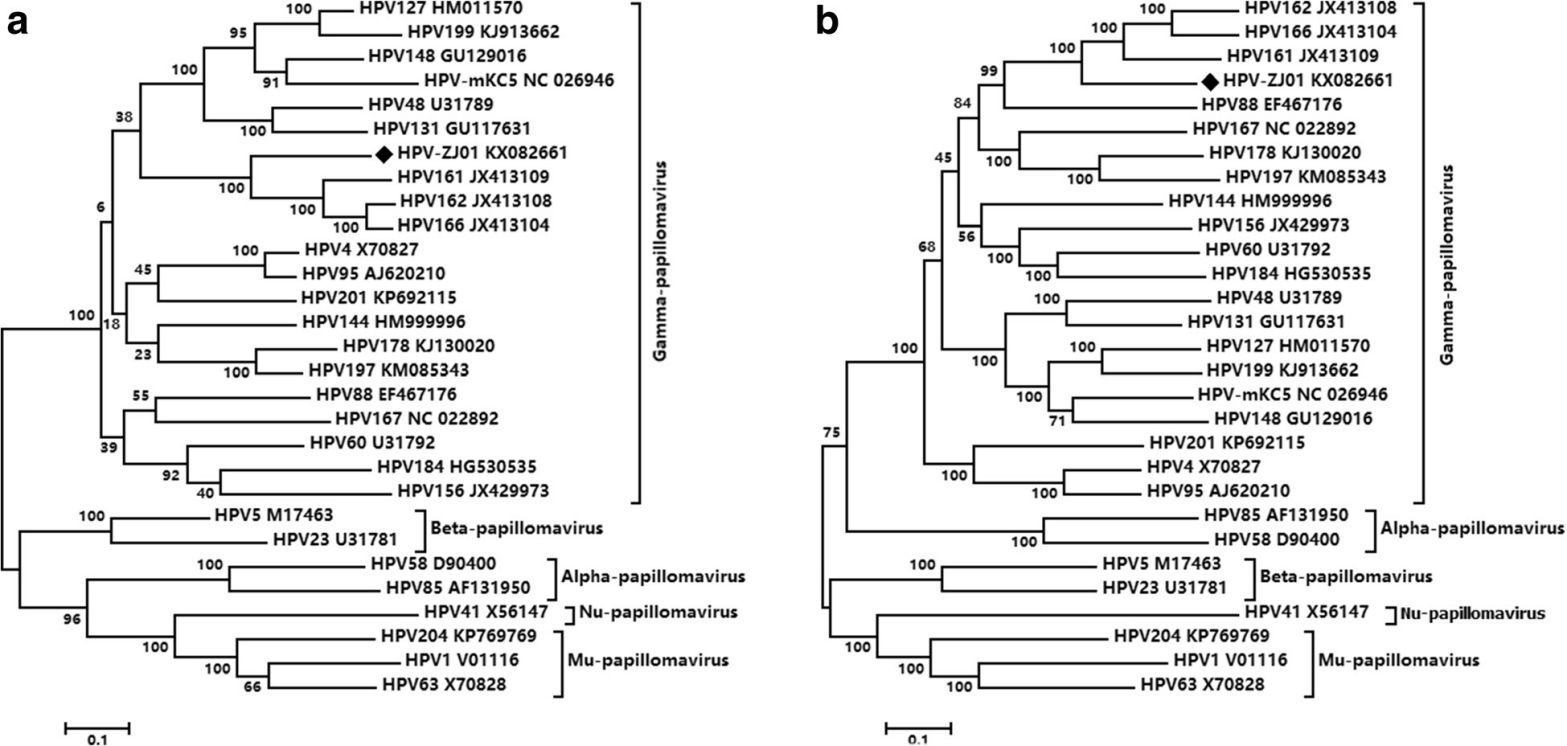
Phylogenetic trees based on the L1 gene (**a**) and complete genome (**b**) of HPV-ZJ01 and other representative human papillomaviruses. The HPV-ZJ01 sequence determined in this study is labeled by black rhombus

To characterize the phylogenetic relationship between HPV-ZJ01 and related HPVs, the entire L1 protein sequence of HPV-ZJ01 and the representative HPV types from other species and genera were aligned to generate a phylogenetic tree, as the L1 gene is well conserved and often used for classification. A phylogenetic tree based on the complete genome of HPV-ZJ01 and the related HPV strains was also constructed to confirm the phylogenetic analysis results based on the L1 protein. Both trees clustered the representative HPVs well in their respective genera, including Gamma-, Alpha-, Beta-, Mu- and Nupapillomavirus, where HPV-ZJ01 clustered within the genus Gammapapillomavirus, species 19 with a bootstrap value of 100 %, forming a monophyletic group with HPV161, HPV162 and HPV166 (Fig. 2a and b).

### PCR screening result

PCR screening results indicated that except the original HPV-ZJ01 positive vaginal swab specimen, none of the other vaginal swabs (n = 135), cervical smears (n = 40) and cervical cancer tissues (n = 40) samples were positive.

## Discussion

The advent of metagenomics using high-throughput sequencing technology [9–12] facilitates the discovery of novel viruses at a relatively low cost and unprecedented speed, assists in the study of viral diversity, and the association of virus and disease [28–30]. We report here the genome of HPV-ZJ01, a novel human gammapapillomavirus type identified from a vaginal swab sample of a pregnant woman, whose L1 gene shares 71.1 % nucleotide identity with that of HPV162, its closest relative. Phylogenetic analysis confirmed that HPV-ZJ01 clusters with other Gammapapillomaviruses in species 19 (HPV161, HPV162 and HPV166), all of them containing the typical HPV ORFs, coding for four putative early proteins (E6, E7, E1, E2), and two late capsid proteins (L2 and L1). Due to the presence of the characteristic donor (AAG/GUASNR) and acceptor (GUYACYAG/YU) RNA splicing sites in E1 and E2 ORFs, respectively [13, 24–26], from HPV-ZJ01, HPV161, HPV162 and HPV166, it is most likely that the E4 proteins of these four HPVs are translated from a spliced mRNA, containing the first few codons of the E1 ORFs joined to the E4 sequence. The resulting putative E1^E4 fusion proteins contain a typically high content of proline, which represents 11 %, 13.27 %, 10 % and 10 % of E1^E4 amino acid sequences of HPV-ZJ01, HPV161, HPV162, and HPV166, respectively.

Analysis of genome organization of HPV-ZJ01, HPV161, HPV162 and HPV166 revealed that they share many similarities and some differences. Two conserved zinc-binding domains of CxxC(x)29CxxC separated by 36 amino acids are found at the same position away from the N-terminal end of E6 protein of these four HPVs, respectively. Additionally, the E7 proteins of them also contain a single zinc-binding domain of CxxC(x)29CxxC at the same position, respectively. The putative LXCXE motif found in the E7 protein of HPV-ZJ01, also identified in the three HPVs of Gamma genus, species 19 is a canonical pRB-binding motif and has been implicated in the transformation of host cells [21, 31, 32]. A homologous LXCXE motif is used to bind to the pocket region of pRB, P107 and P130 and prevent inter-actions with the transcription factor E2F-1 by most HPV E7 proteins [33]. The E1 of HPV-ZJ01 contains the conserved ATP-binding site of the ATP-dependent helicase (GPPDSGKS), nevertheless, in comparison, the motif is GPPDTGKS in all of the other three HPVs, with the Serine being replaced by Threonine. The putative E2 protein of HPV-ZJ01 contains no conserved leucine-zipper domain (L-X6-L-X6-L-X6-L), necessary for E2 dimerization. This domain is also missing in HPV161, 162 and 166. In the LCR of both HPV-ZJ01 and HPV161, a putative TATA box (TATAAA) of the E6 promoter is identified 36 and 34 nt upstream of the first start codon of the E6 ORF, respectively, while the classical TATA box (TATAAA) was not detected in the LCR of HPV162 and HPV166. Similarly, three and two E1 binding sites (AACAAT) are found in the LCR of HPV-ZJ01 and HPV161, respectively, while none is detected in the LCR of HPV162 and 166. As for the polyadenylation sites, the LCR of HPV-ZJ01 and HPV162 each contain one and HPV161 and HPV166 each contain two. Notably, for HPV161, its LCR simply contains two copies of the 12-basepair palindrome motif ACCG-N4-CGGT, known to play a crucial role in binding of the E2 protein [18, 19]. However, in the other three HPVs (HPV-ZJ01, HPV162, HPV166), both palindrome sites (ACCG-N4-CGGT) and degenerate palindrome sites (ACC-N6/7-GGT) are simultaneously present in their LCRs. HPV-ZJ01 contains two palindrome sites (ACCG-N4-CGGT) and three degenerate palindrome sites (ACC-N6/7-GGT) in its LCR, while the LCR of HPV162 only contains one palindrome site (ACCG-N4-CGGT), and the other three E2 binding sites are represented by the degenerate ones (ACC-N6-GGT). Only one palindrome site (ACCG-N4-CGGT) and four degenerate palindrome sites (ACC-N6-GGT) are in the LCR of HPV166.

HPV types have traditionally been classified as mucosal or cutaneous on the assumption that HPV tissue tropism is reflected in the location where the genome of the particular type was found [13, 34]. Accordingly, HPV types belonging to the Alpha-PVs are predominantly assigned as anogenital types and Beta-PVs, Gamma-PVs, Mu-PVs and Nu-PVs as cutaneous types [2, 34]. However, a growing body of evidence suggests that HPV types belonging to Gamma-PVs (the genus whose known members has recently been expanding most rapidly) are ubiquitous and show broader tissue tropism than previously thought [13], with reported detection sites ranging from healthy skin and various cutaneous lesions [35–38] to genital lesions [39] and oral [34] and nasal [40] mucosa. One recent report suggests that Gamma-PVs are also able to infect mucocutaneous sites such as the anal canal [41] and even the possibility of dual tropism of Gamma HPVs was recently proposed [26]. Here, HPV-ZJ01, confirmed to be a member of Gamma-PV genus, species 19 and identified in a vaginal swab can be preliminarily classified as mucosal. However, as its closest relatives (HPV161, HPV162 and HPV166) were isolated from healthy skin [27], the tropism of HPV-ZJ01 remains to be definitely established as different PV types classified in a specific species typically share biological and pathological properties [3]. The 25-year-old pregnant woman in whom we identified HPV-ZJ01 was suffering from vaginitis. Whether the presence of HPV-ZJ01 was at all related to this condition or indirectly facilitated viral replication is not known. No skin or mucosal lesion was noted by the attending gynecologist. In humans, cutaneous HPVs are highly prevalent in the general population, where they usually are contained by the immune system and result in subclinical infections. These infections however tend to become clinically apparent upon genetic, acquired, or iatrogenic deficiencies in cell-mediated immunity, and cutaneous HPVs have been linked to non-melanoma skin cancer in these patients [42].

Here we screened total 216 samples including vaginal swabs, cervical carcinoma tissues and cervical smears from cervical carcinoma patients using PCR methods. The negative PCR results suggested that HPV-ZJ01 may not be associated with cervical cancer. Further studies with different sample types and a larger sample size are needed to clarify the prevalence and disease association, if any, for HPV-ZJ01.

## Conclusion

A novel HPV type named HPV-ZJ01 was identified in a vaginal swab sample from a 25 years old pregnant woman with vaginitis and its genome was characterized. Comparative characterization of HPV-ZJ01 and its closest relatives and phylogenetic analysis demonstrated that it is a novel HPV type and clusters into the Gamma-PV genus, species Gamma-19. No evidence of disease association of the new HPV type was found.

## Abbreviations

Alpha-PVs, Alphapapillomavirus; Beta-PVs, Betapapillomavirus; DNase, deoxyribonuclease; Gamma-PVs, Gammapapillomavirus; HPV, human papillomavirus; ICTV, International Committee on Taxonomy of Viruses; LCR, non-coding long control region; Mu-PVs, Mupapillomavirus; NCBI, National Center for Biotechnology Information; nt, nucleotide; Nu-PVs, Nupapillomavirus; ORF, open reading frame; PBS, phosphate-buffered saline; PCR, polymerase chain reaction; PV, papillomavirus; RCA, rolling circle amplification; RNase, Ribonuclease; URR, upstream regulatory region.
